# Network Pharmacology-Based Strategy to Investigate Pharmacological Mechanisms of Qiaoshao Formula for Treatment of Premature Ejaculation

**DOI:** 10.1155/2020/1418634

**Published:** 2020-11-11

**Authors:** Ming Wang, Qi Wang, Yongqiang Du, Xiansheng Zhang

**Affiliations:** ^1^Fuyang People's Hospital, Anhui Medical University, Fuyang 236000, Anhui, China; ^2^Renji Hospital, Shanghai Jiao Tong University School of Medicine, Shanghai 200127, China; ^3^Department of Urology, First Affiliated Hospital of Anhui Medical University, Hefei 230022, Anhui, China

## Abstract

**Background:**

Qiaoshao (QS) formula, a traditional Chinese medicine (TCM) comprising seven herbs, has been clinically proven to have a favorable treatment effect on premature ejaculation (PE). However, its underlying pharmacological mechanisms in the treatment of PE need to be further clarified.

**Methods:**

In the present study, a network pharmacology-based strategy was adopted. The active compounds of QS formula were obtained from the Chinese medicine database, and the potential targets of these compounds were collected from the DrugBank database to construct compound-compound targets network. PE-related targets were identified from human disease databases and used to construct the protein-protein interaction (PPI) networks. Compound-disease target PPI network was constructed by merging the PPI network of disease-targets and compound-targets. Cluster and enrichment analyses were performed on the PPI network of disease targets and compound-disease targets. The influence of QS formula on serum 5-HT, NO, oxytocin, and thyroid hormones of PE patients was verified.

**Results:**

Four primary pharmacological networks of QS formula were constructed, including the compound-compound targets network, PPI network of PE-related targets and compound-disease targets, and the QS-PE mechanism network. The module and pathway enrichment analyses revealed that the QS formula had the potential to affect varieties of biological process and pathways, such as nitric oxide biosynthetic process, oxytocin, thyroid hormone, TNF, PI3K-Akt, and the HIF-1 signaling pathway, that play an important role in the pathogenesis of PE. Meanwhile, the QS formula has been clinically confirmed to regulate the serum level of 5-HT, NO, oxytocin, and TT in PE patients.

**Conclusion:**

This study preliminarily discovered the potential targets and pathways of QS formula in the treatment of PE, which laid a good foundation for further experimental research.

## 1. Background

Premature ejaculation (PE) is the most common form of male sexual dysfunction with prevalence rates of 20–30% [1]. According to the European Society of Surgeons (ESS), PE is defined as the intravaginal ejaculatory latency time (IELT) less than 1  minute (primary PE), or the former is shortened to less than 3 minutes (secondary PE), accompanied by the inability to delay ejaculation and mental health problems [2]. At present, the etiology of PE is still unclear. The mainstream view is that it may be associated with some physiological factors, including serotonin [3], penile hypersensitivity [4], hormones [5], genetic variation [6], prostatitis, thyroid, and other psychological factors [8, 9]. Although PE is not life-threatening, it has serious psychological influences on PE patients, leading to mental distress, anxiety, and depression, which in turn affects the quality of life of patients and their partners.

The treatment of PE needs the implementation of personalized comprehensive treatment according to the specific etiology. At present, treatments for PE mainly include drug therapy, behavioral psychotherapy, surgical treatment, and traditional Chinese medicine (TCM) [10]. Qiaoshao (QS) formula is clinically used to treat lifelong PE (LPE) and can improve satisfaction in sex life. It contains seven Chinese herbal medicines, including Forsythiae Fructus (FF), Paeoniae Radix Alba (PR), Radix Bupleuri (RB), Hedysarum multijugum Maxim. (HM), Morindae officinalis Radix (MO), Rhizoma Dioscoreae (RD), and Acoritataninowii Rhizoma (AR). Previous pharmacology research of TCM has been primarily focused on single targets or a few pathways [11, 12], which resulted in a lack of acknowledgment of the synergy between multicomponent, multiple targets, and multipathways in disease treatment of TCM. Currently, the precise molecular mechanisms of QS formula in the treatment of PE remain unclear.

**Figure 1:**
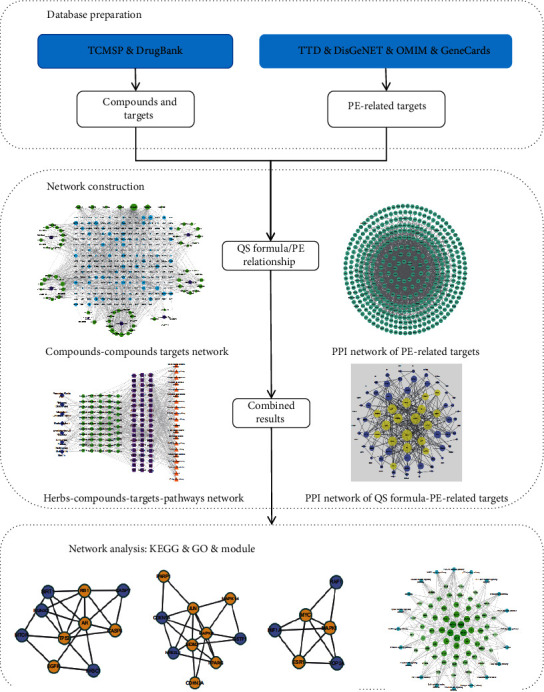
Workflow for Qiaoshao formula in the treatment of premature ejaculation.

Network pharmacology with systematic and holistic characteristics has been developed in recent years to explore the intricate mechanisms of TCM and extensively applied by TCM researchers [13, 14]. To provide evidence for the further in-depth development of the basic experimental research and clinical application of QS formula in PE treatment, we aim to use the network pharmacology to explore the potential mechanisms of how QS formula exerts the therapeutic effects on PE. The flowchart of network pharmacology-based study of QS formula is shown in Figure 1.

## 2. Materials and Methods

### 2.1. Patients

Forty-one married men (aged 22–50) with PE and forty-one healthy men (aged 24–49) as the control group were included in this study. PE was evaluated by intravaginal ejaculation latency time (IELT), while IELT < 1 min was accepted PE. Exclusion criteria of the study were patients with diabetes mellitus, chronic disorders, or erectile dysfunction, heavy smokers, and patients with major psychiatric or psychological illness. All the patients received QS formula twice a day for 4 weeks, and all patients were required to have one intercourse episode per week. Committee of ethics approved the protocol, and all participants provided written informed consent.

### 2.2. Blood Detection

Blood samples were obtained in the morning after an overnight fasting. Total testosterone (TT), follicle-stimulating hormone (FSH), luteinising hormone (LH), and prolactin levels were investigated with chemiluminescent immunoassay. The serum oxytocin and serotonin (5-HT) levels were measured using an enzyme-linked immunosorbent assay (ELISA). Production of nitric oxide (NO) was determined by measuring accumulation of nitrite in serum using the Griess reaction with sodium nitrate as the standard. In brief, 50 *μ*l samples of serum were mixed with equal volumes of 1 percent sulphanilamide and 0.1 percent *N*-(1-naphthyl) ethylenediamine dihydrochloride in 0.5 percent H_3_PO_4_. After 10 min at room temperature, the absorbance at 540 nm was measured in a microplate reader [15].

### 2.3. Active Ingredients Screening and the Targets

The chemical composition of the seven herbs in the QS formula was retrieved from the Traditional Chinese Medicine Systems Pharmacology Database and Analysis Platform [10] (TCMSP, http://lsp.nwu.edu.cn/tcmsp.php). The candidate compounds which have oral bioavailability (OB) ≥40% and drug-likeness (DL) ≥ 0.2 were identified as active compounds [16]. A bioactive molecule with high OB displays good DL, which is a qualitative concept utilized in drug design to optimize pharmaceutical and pharmacokinetic properties of molecules, such as chemical stability and solubility [17]. In addition, the targets of candidate compounds were obtained from the TCMSP database. The candidate compounds were then imported into the DrugBank database (https://www.drugbank.ca/) to identify the targets.

### 2.4. Searching Targets of PE Disease

PE-related targets were retrieved from the following four existing resources: (1) Therapeutic Target Database (TTD, http://db.idrblab.net/ttd), (2) DisGeNET (https://www.disgenet.org/), (3) Online Mendelian Inheritance in Man (OMIM, https://www.omim.org/), and (4) GeneCards (https://www.genecards.org/), using the keyword “premature ejaculation” to search. The disease targets obtained from the above databases were pooled to identify targets related to PE.

### 2.5. Network Construction

#### 2.5.1. Method for Network Construction

In order to demonstrate the mechanism of action of the QS formula in the treatment of PE, several dominating networks were constructed, including the disease PPI network, the drug compounds and compound-target network (C-CT network), the drug compound-disease target network (C-DT network), and the drug compounds disease target-pathway network (C-DT-P network), using the Cytoscape software (version 3.7.1; https://cytoscape.org/download.html) [18]. It can help identify the primary mechanism of complex TCM in the treatment of diverse diseases. The PPI data were obtained using the Bisogenet plugin in Cytoscape software. Only the interactions between the input nodes were retrieved and presented in the PPI network [19, 20]. All self-loop interactions were removed to show PPI directly and concisely.

#### 2.5.2. Bioinformatic Analysis and Module Analysis

GO and KEGG enrichment analyses were carried out using the Database for Annotation, Visualization and Integrated Discovery (DAVID, https://david.ncifcrf.gov/ v6.8) [21]. Enrichment analysis results of the study were screened at FDR < 0.05, and the specific disease pathways were excluded [22]. Molecular complex detection (MCODE) algorithms can find dense regions of interaction in PPI networks based on complex connection data [23]. We use the MCODE plugin for Cytoscape software to carry out module analysis on protein targets in the PPI networks according to the default parameters [23, 24].

### 2.6. Statistics Analysis

The Statistical Package for Social Sciences (SPSS 12.0.1; SPSS Inc., Chicago, Ill., USA) was adopted to perform statistics analysis. The results were compared with independent-samples *t* tests and paired-samples *t* tests for statistical analysis. *P* < 0.05 was accepted as the statistically significant value.

## 3. Results

### 3.1. Drug C-T Network Analysis

#### 3.1.1. Active Compounds Screening of QSD

A total of 100 active compounds in the QS formula were identified based on the OB and DL parameters, including AR with 4 compounds, FF with 23 compounds, HM with 20 compounds, MO with 20 compounds, PR with 13 compounds, RB with 17 compounds, and RD with 16 compounds. Detailed information on the chemical constituents of each herb and the chemical parameters of the active compounds in QS formula is described in Table S1.

#### 3.1.2. Drug Compound-Compound Target Network

Twenty-four of the 100 potentially active compounds without any human targets were removed, and the remaining 76 active compounds with 252 targets were used to construct the C-CT network. The established C-CT network consisted of 335 nodes and 1185 edges, including the seven herbs contained in QS formula, 76 active compounds, and 252 targets constituting the network node. These potentially active ingredients produced a total of 1110 interactions with targets. The number of active ingredients and targets of the seven herbs that were contained in the QS formula Chinese medicine compound in the C-CT network are as follows: MO contains sixteen potentially active ingredients that act on 55 targets, AR includes four potentially active compounds that correspond to 78 targets, HM contains 17 potentially active ingredients and 206 targets, PR includes 8 potentially active ingredients with 81 targets, RD contains 12 potentially active ingredients with 76 targets, RB includes 13 potentially active compounds that correspond to 189 targets, and FF contains 19 potentially active ingredients with 220 targets (Table 1).

**Figure 2:**
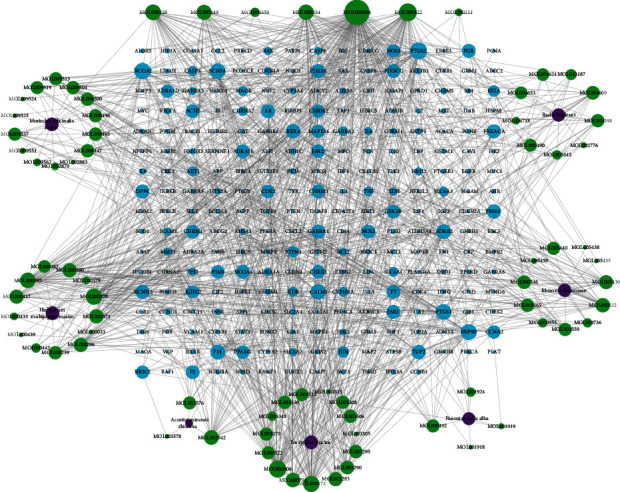
Qiaoshao formula compound-compound target network (purple quadrilaterals, green circles, and blue triangles represent herbs, compounds, and targets, respectively).

In this C-CT network, the interactions of the nodes were not in equilibrium. Some compounds can regulate multiple targets (for example, quercetin compounds can act on 147 potential targets, kaempferol can regulate 60 targets, and luteolin can affect 57 potential targets). Conversely, some ingredients can only affect a few targets, for instance, cycloartenol, which only affects NR3C protein. Similarly, some targets can be synergistically coordinated by multiple chemical components, while others are only affected by few compounds. For example, the PTGS2 target can be regulated by 50 compounds, and NCOA2 protein can be regulated by 43 compounds, but some proteins such as CTRB1 and MAOA are regulated by only one compound. The uneven distribution of compound-target network nodes indicated that QS formula treatment may rely on the role of some key compounds. The network details are described in Figure 2 and Table S2.

### 3.2. Disease Target Network Analysis

#### 3.2.1. PE Disease-Related Targets

**Figure 3:**
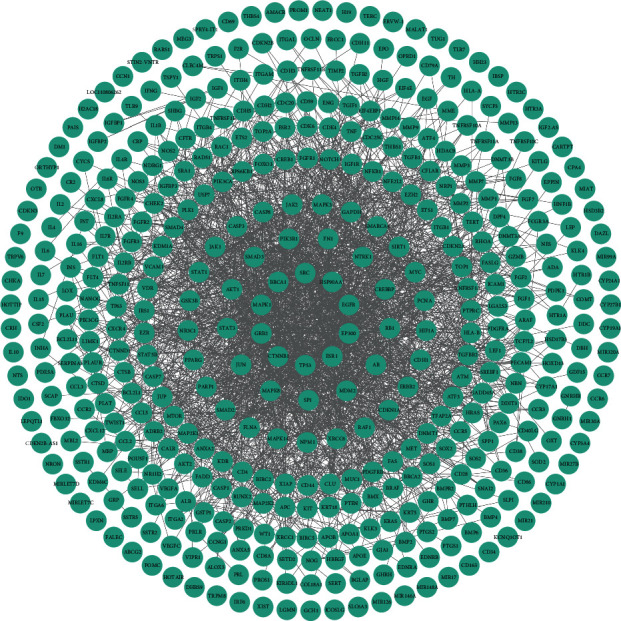
PPI network of PE-related targets.

From the TTD database, we obtained 5 PE-related proteins, 5 from the OMIM database, 418 targets from the GeneCards database, and 2 from the DisGeNET system. A total of 425 PE disease-related targets were obtained (Table S3), and a PPI network of these targets was constructed (Figure 3). The disease PPI network consisted of 425 nodes and 2197 edges. The closer the node to the center of the network, the higher the degree. Higher degree of a node in the network suggested it is more likely to play an important role in disease development. The protein nodes in the center of PPI network, such as TP53, HSP90AA1, EP300, EGFR, SRC, and MAPK1, have higher degrees, which corresponded to a degree of 71, 69, 66, 65, 64, and 62, respectively. Therefore, they may be closely linked to PE development. Network details are described in Table S4.

#### 3.2.2. Module Analysis of the Disease PPI Network

The PPI network was clustered by module analysis using the MCODE package, and three clusters with score > 4 were used to analyze the mechanism of PE disease (see Table 2 for each type of clustering target and Figures S1–S3 for clustering target relationship). By analyzing the relevant biological processes, molecular functions, and cellular components of every cluster, we found that PE disease was primarily related to the regulation of cell proliferation, gene expression, apoptotic process, immune response, inflammatory response, and other processes. Details of GO enrichment analysis of disease targets and each module are described in Tables S5–S8.

#### 3.2.3. Pathway of the PE Network

PE disease-related proteins were put into DAVID for pathway analysis, and 43 pathways were acquired after deleting results for specific disease pathways. The top 5 enriched pathways including the PI3K-Akt signaling pathway contains 89 proteins, the FoxO signaling pathway contains 52 proteins, 34 proteins were enriched in the Prolactin signaling pathway, and 37 proteins were enriched into the TNF signaling pathway; Rap1 signaling pathway contains 50 proteins. The study obtained a pathway of the PE network by aggregating the disease PPI network and PE-related protein pathway analysis. The network directly revealed interactions between disease proteins and the relationship of disease-related pathways. The network consists of 291 nodes (43 pathways and 248 PE-related proteins) and 1306 edges. Detailed information on the PE disease pathway is provided in Figure S4 and Table S9.

### 3.3. QS Formula-PE Network Analysis

**Figure 4:**
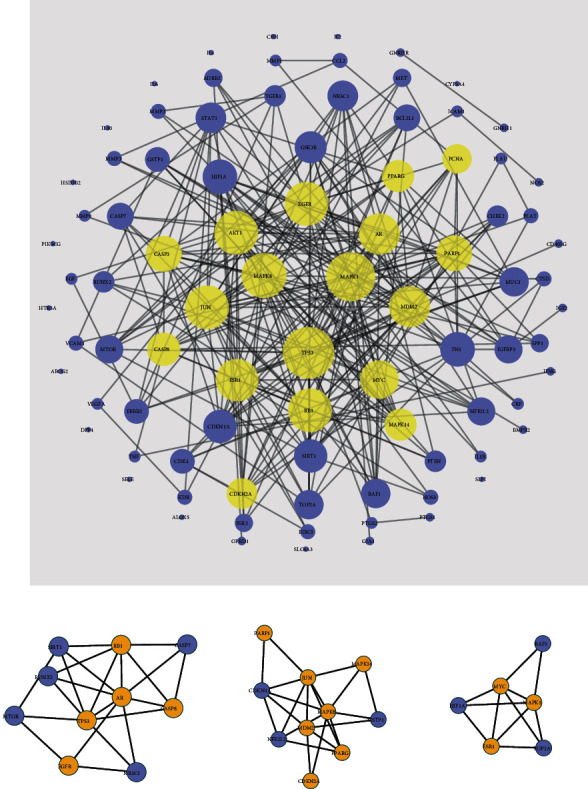
PPI network of QS formula-PE targets and modules: (a) PPI network of QS formula-PE targets; (b, d) module analysis of PPI network of QS formula-PE targets. Yellow nodes represent key targets.

QS formula-PE disease target network (C-DT network) was acquired by combining the C-CT network with the disease PPI network. Nodes of herbs and compounds were hidden. The C-DT network succinctly demonstrates the interaction between each active compound in the QS formula and PE disease targets, and multicomponent multiple target interactions are presented in a network map. The C-DT network is composed of 88 nodes and 280 edges. Using a Cytoscape plugin (CytoNCA), we selected the following seven topological features to identify candidate targets: betweenness centrality (BC), degree centrality (DC), closeness centrality (CC), eigenvector centrality (EC), network centrality (NC), and local average connectivity (LAC) [25]. The median values of BC, CC, DC, EC, LAC, and NC were 0.018, 0.35, 6, 0.071, 1.927, and 2.828, respectively. Thus, we identified 18 key targets with values for these topological features higher than the reported median values and represented by yellow circles in Figure 4(a).

### 3.4. Module Analysis and Functional Enrichment Analysis

A network module or cluster is defined as a highly interconnected set of nodes that help discover and reveal hidden biological information within the network. In order to identify the potential mechanism of the QS formula-PE targets, the QS formula-PE PPI network was divided into 3 clusters (Figure 4(b) and Table 3). And a total of 15 key targets (MYC, ESR1, MAPK1, JUN, PARP1, MAPK8, MAPK14, CDKN2A, PPARG, MDM2, RB1, CASP8, EGFR, TP53, and AR) were clustered in these 3 modules.

Next, we performed GO enrichment analysis of the QS formula-PE targets to gain insights into the cellular component (CC), molecular function (MF), and biological processes (BP) that are affected in PE (Table S10). The results indicated the potential targets were highly connected with the regulation of transcription, apoptotic process, cell proliferation, protein phosphorylation, and nitric oxide biosynthetic process.

Furthermore, KEGG pathway enrichment analysis was carried out for the QS formula-PE targets (Table S11). The results demonstrated that QS formula-PE targets were highly correlated with signal transduction, immune system, cardiovascular disease, and infectious disease. We recognized 43 PE-related signaling pathways, including the TNF signaling pathway, the PI3K-Akt signaling pathway, and the HIF-1 signaling pathway. Therefore, the results imply that QS formula treats PE by participating in above biological process and signaling pathway.

### 3.5. YGL Capsule C-DT-P Network

**Figure 5:**
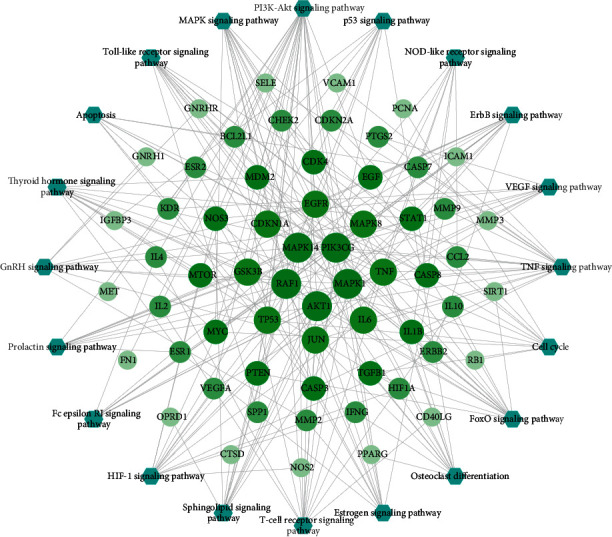
Pathways of the QS formula-PE targets network. Blue hexagons and green circles represent pathways and targets, respectively.

The direct and indirect action targets of QS formula were placed in the DAVID database to analyze action pathways. Specific disease pathways were deleted from the results, leaving 20 pathways. The relationships between these proteins and pathways were placed in Cytoscape to construct the pathway of the QS formula-PE network (Figure 5). Details of topological characteristics of the QS formula-PE network are provided in Table S12. Pathways of the QS formula-PE network only include intersecting target networks that were obtained by merging the pathway of the QS formula-HB network with the QS formula-HB disease targets network. This network consisted of 81 nodes (20 pathways and 60 related proteins), and the relationship between the nodes produced 245 edges. In these 20 pathways, the PI3K-Akt pathway was enriched by 24 proteins and had the highest degree of enrichment; the TNF signaling pathway was enriched by 19 proteins; the FoxO signaling pathway was enriched by 15 proteins; MAPK, *T*-cell receptor, and HIF-1 signaling pathways were enriched by 14 proteins; the ErbB signaling pathway was enriched by 13 proteins; sphingolipid, Toll-like receptor, thyroid hormone signaling pathway, and osteoclast differentiation were enriched by 12 proteins; and the cell cycle and estrogen signaling pathway were enriched by 11 proteins. These pathways may represent the primary mechanisms for how QS formula ameliorates PE disease.

**Figure 6:**
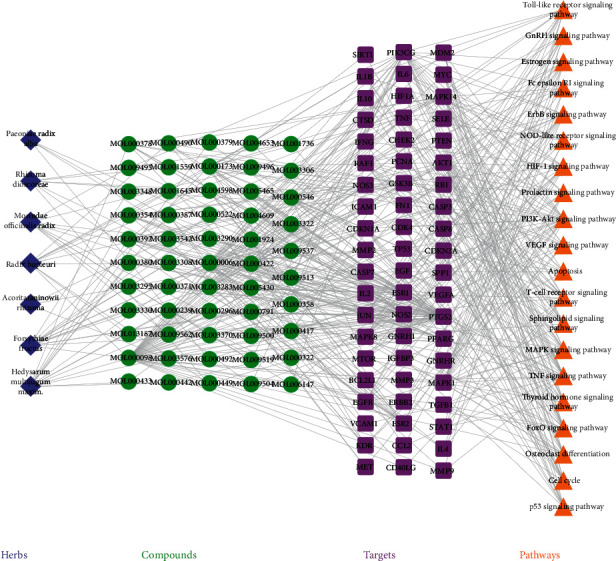
Herbs-compounds-targets-pathway network (purple indicates herbs, green indicates compounds, pink indicates targets, and orange indicates pathways).

**Figure 7:**
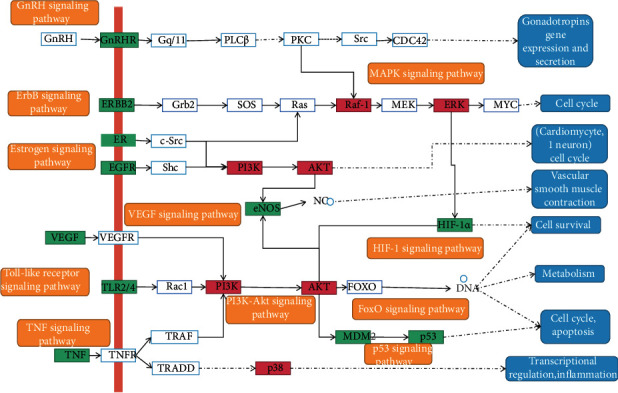
Integrated pathways involved in the treatment of QS formula for PE patients (key targets located at the center of pathway-targets network were colored in red, other targets of QS formula were colored in green, and other protein targets in the pathway were not colored; the software of PowerPoint was used to generate the figure).

The C-DT-P network could clearly define relationships between herbs, targets, and targets and pathways but lacked the ability to demonstrate relationships between herbs and compounds. Additionally, due to a large number of nodes and the relationships between the networks, the network is still relatively complicated. To succinctly show the main mechanism of action of QS formula, the H-C-DT-P network and C-DT-P network were merged to obtain the H-C-DT-P network. Targets with linked pathways and compounds with linked targets were retained, and the final H-C-DT-P network consisted of seven herbs, 54 compounds, 61 targets, and 20 pathways. Detailed information is provided in Figure 6. In addition, an integrated pathway presenting the crosstalk among enriched pathways was constructed (Figure 7).

### 3.6. Clinical Validation

To further validate the finding from network pharmacology, the influences of QS formula on the serum concentration of 5-HT, NO, oxytocin, and thyroid hormones in PE patients were detected, as shown in Table 4. Compared with the control group, 5-HT and NO levels were statistically lower in patients with PE, while oxytocin and TT levels were statistically higher in patients with PE (*P* < 0.05). These were consistent with previous observations [15, 26, 27]. There was no significant difference in the levels of FSH, LH, TSH, and prolactin between the two groups. After treatment with QS formula, 5-HT and NO levels were increased from baseline (5-HT: 75.84 TO 118.69 and NO: 26.89 TO 31.59) (*P* < 0.05), whereas oxytocin and TT levels were decreased from baseline (oxytocin: 98.79 to 71.01 and TT: 5.04 to 3.82) (*P* < 0.05), suggesting that QS formula could regulate the serum 5-HT, NO, oxytocin, and TT in PE patients.

## 4. Discussion

It is currently believed that PE may be caused by (1) psychological reasons: self-confidence and uneasiness; (2) organic causes: penile hypersensitivity and increased sensory nerve excitability; (3) others: inflammatory diseases, sympathetic ganglia damage, polycythemia, and drug withdrawal syndrome. In this study, the module and enrichment analyses of PE-related targets demonstrated that diverse biological processes were involved in the development of PE, such as transcription regulation, cell proliferation and apoptosis, and signal transduction. The enriched pathways of PE-related targets mainly included 44 pathways, such as PI3K-Akt, TNF, FoxO, and MAPK signaling pathway. Some targets (e.g., MAP2K1, MAP2K2, AKT1, PIK3CG, AKT2, PIK3CA, MAPK1, MAPK3, KRAS, RAF1, PIK3R1, and HRAS) located at the center of the disease-pathway network act on various pathways, which may hold an important role in the pathogenesis of PE and may be potential targets for future PE treatment.

By merging the C-CT network of QS formula and the PE-targets PPI network, we obtained 88 anti-PE targets from QS formula, and then, module analysis and enrichment analysis were performed to further investigate the potential mechanism. It revealed that 20 significant enriched pathways were associated with QS formula in the treatment of PE disease, including TNF, PI3K-Akt, HIF-1, *T*-cell receptor, ErbB, FoxO, Toll-like receptor, p53, thyroid hormone, prolactin, sphingolipid, estrogen, VEGF, NOD-like receptor, MAPK, and Fc epsilon RI signaling pathway, as well as osteoclast differentiation, cell cycle, and apoptosis. These pathways participate in many important biological processes, such as signal transduction, regulation of cell proliferation and gene expression, cellular response to mechanical stimulus, protein phosphorylation, and nitric oxide biosynthetic process.

It is necessary for the occurrence of ejaculation to synergistically activate the autonomic and somatic nervous systems. It has been demonstrated that the sympathetic nervous system was able to command the contractile activity of sex glands and related ducts [26, 27]. Synapse is an important part of the nervous system, and many pathways involved in modulating synaptic plasticity, such as TNF [28], PI3K-Akt [29], HIF-1, and *T*-cell receptor signaling pathway [30]. The ErbB protein family or EGFR family is a family of four structurally related receptor tyrosine kinases. Insufficient ErbB signaling in humans is related to the development of some neurodegenerative diseases, such as multiple sclerosis and Alzheimer's disease [31]. Recently, the crucial role of the Nrg-1/ErbB network in neurodevelopment has also been identified [32]. The FOXO family is evolutionarily conserved and a significant arbiter of neural cell fate and function in mammals. Under both physiological and pathological conditions, it could modulate neural cell survival, stress responses, lineage commitment, and neuronal signaling in the process of the neural stem cell to mature neurons [33]. The opioid analgesic tramadol has been reported to be an effective on-demand treatment for PE patients [34]. The activation of Toll-like receptor-4 could trigger signal transmission pathways in the nervous system, leading to chronic pain as well as opioid tolerance and dependence [35]. The p53 protein is a nuclear transcription factor that regulates the expression of a wide variety of genes involved in diverse biological processes and prevents neurodegeneration by regulating synaptic genes [36]. Therefore, the underlying mechanism of QS in the treatment of PE might be to participate in the management of ejaculation in the nervous system by regulating these crucial pathways.

It has attracted great attention with respect to the role of cerebral serotonin in the control of the ejaculatory response. Oral intake of selective serotonin reuptake inhibitors appeared effective for patients with PE [37]. In this study, we observed several PE-related targets were enriched in the serotonin binding, regulation of serotonin secretion, and adenylate cyclase-inhibiting serotonin receptor signaling pathway, confirming the crucial role of serotonin in the development of PE. Pharmacological studies have demonstrated that the effect of herbs in QS formula was related to serotonin. The active component of Fructus Forsythiae is St John's wort, which could inhibit the uptake of serotonin, dopamine, and norepinephrine and delay the rapid ejaculation induced by serotonin agonists in a rat model [38]. Morinda officinalis has been proved to decrease the serum level of serotonin [39]. It was reported that the combination use of Radix Bupleuri and Radix Paeoniae Alba could increase serotonin, dopamine, and norepinephrine significantly in a chronic stress rat model [40]. In addition, the level serotonin level in the brain of rats could be increased by Rhizoma Acori Graminei [41]. Clinically, the influences of QS formula on serum serotonin of PE patients have also been confirmed in this study.

Nonadrenergic noncholinergic (NANC) innervation participates in the control of ejaculation by regulating the accessory sex glands activity. In the NANC autonomic system, nitric oxide (NO) fiber is the main component and has been observed in the entire seminal tract of human [42]. In addition, the activity of smooth muscle contractile could be reduced by stimulating the NO intracellular signaling pathway in seminal vesicles [43]. In this study, GO annotation results demonstrated the QS formula-PE targets were enriched in the nitric oxide biosynthetic process, and clinical validation also showed that QS formula affects the serum concentration of NO in PE patients. Several genes may play an important role in the process, including AKT1, EGFR, ESR1, ICAM1, IFNG, IL1B, IL6, MTOR, PTGS2, and TNF. Nitric oxide synthesis by endothelial nitric oxide synthase is precisely regulated by protein kinases including AKT1 [44], and the quercetin from three herbs of QS formula has been revealed to suppress the protein level of pAKT1 [45].

Hormones are dramatically participants in the ejaculatory mechanism [46]. Oxytocin is a neurohypophysial hormone and involves in the regulation of contractility of the male genital tract in some animal species. It has been suggested that oxytocin would play a key role in the central regulation of penile erection as well as in the ejaculatory process, by regulating semen emission [47] and penile detumescence [48]. Specific oxytocin receptors (OTR) have been discovered in the tunica albuginea, epididymis, and vas deferens [49]. OTR in the epididymis was revealed to trigger the release of endothelin to probably amplify the OX-induced contraction [50]. KEGG enrichment analysis showed that 16 PE-related targets were involved in the oxytocin signaling pathway, and 7 compounds targets of QS formula were involved in the oxytocin signaling pathway, suggesting the potential importance of these 7 targets of QS formula in the treatment of PE. In addition, QS formula showed the ability to regulate the serum level of oxytocin in PE patients in this study. The seven targets were JUN, RAF1, CDKN1A, EGFR, MAPK1, NOS3, and PTGS2, respectively. It was reported that the OTR-coupled MAPK-MEF-2A pathway was responsible for OT-induced neurite retraction of hypothalamic neurons [51] and OTR could transactivate EGFR to the different temporal patterns of EGFR [52].

Moreover, thyroid status extremely influences mood and relational life, and patients with thyroid disorders can have various sexual symptoms. It was confirmed that hyperthyroidism affects both the emission and expulsion phases of ejaculation, whereas hypothyroid delayed ejaculation by increasing the latency and decreasing the number and frequency of bulbospongiosus muscle contractions [47]. Both ejaculatory dysfunctions reverted after euthyroidism was achieved without any other treatment for the sexual symptom. This suggests that thyroid hormones directly involved in the physiology of ejaculation. KEGG enrichment analysis of QS formula-PE targets showed that 12 compound targets were enriched in the thyroid hormone signaling pathway, suggesting the potential role of these targets of QS formula in the treatment of PE. Moreover, for PE patients, QS formula decreased the serum level of TT, whereas it has no effect on FSH, LH, TSH, and prolactin. Therefore, the potential mechanism of QS formula in PE is related to the regulation of serum 5-HT, nitric oxide, oxytocin, and TT.

## 5. Conclusion

In the present work, we applied a network pharmacology approach to holistically decipher that the pharmacological mechanisms of QS formula in the treatment of PE. The active compounds of QS formula and their targets and PE-related targets were identified, and the networks were constructed to show the interactions among them. The potential mechanisms of QS formula in the treatment of PE may be associated with its involvement into the regulation of signal transduction, 5-HT, nitric oxide, and hormones. Clinically, QS formula has also been validated to regulate the concentration of serum 5-HT, nitric oxide, oxytocin, and TT. However, further experimental experiments were required to discover the deeper molecular mechanisms behind these regulations on the basis of above network pharmacology findings.

## Figures and Tables

**Table 1:** Number of potentially active compounds per herb and the number of targets.

Herbal name	Potential active compound number	Targets number
MO	16	55
AR	4	78
HM	17	206
PR	8	81
RD	12	76
RB	13	189
FF	19	220

FF: Forsythiae Fructus; PR: Paeoniae Radix Alba; RB: Radix Bupleuri; HM: Hedysarum multijugum Maxim.; MO: Morindae officinalis Radix; RD: Rhizoma Dioscoreae; AR: Acoritataninowii Rhizoma.

**Table 2:** PE disease PPI network cluster analysis.

Cluster	Scores	Nodes	Edges	Node
1	11.857	15	83	PARP1, HIF1A, MYC, BRCA1, SMAD2, CREBBP, SP1, MAPK1, SMAD4, MAPK3, SMAD3, TP53, EP300, ESR2, CTNNB1
2	7.267	31	109	FAS, GAPDH, PDGFRB, SMARCA4, PDGFRA, RUNX2, EGFR, JUN, JUP, CASP8, STAT5B, GADD45A, IGF1R, EZR, ESR1, STAT1, AKT1, NFKB1, XRCC6, FADD, TNFRSF1A, VDR, ERBB2, RHOA, NR3C1, MAPK14, ETS1, FOXO3, AR, PPARG, FASLG
3	6.786	29	95	ANXA2, KDM1A, SIRT1, MAP2K2, BRCA2, MAPK8, BRAF, BMX, JAK2, JAK1, STAT3, SRC, TERT, CXCR4, CDKN1A, GHR, PIK3R1, PIK3CA, RB1, RAF1, PTPRC, EZH2, FLNA, MUC1, CCR5, SOS2, SOS1, NTRK1, GRB2

**Table 3:** Clusters within the QS formula-PE PPI network.

Cluster	Score	Node	Edges	Node
1	4.889	10	22	CASP8, RB1, CASP7, RUNX2, NR3C1, MTOR, EGFR, AR, SIRT1, TP53
2	4.667	10	21	JUN, NFE2L2, MDM2, MAPK8, MAPK14, PARP1, CDKN1A, PPARG, GSTP1, CDKN2A
3	4.400	6	11	MYC, MAPK1, TOP2A, HIF1A, RAF1, ESR1

**Table 4:** 5-HT, NO, oxytocin, prolactin, FSH, LH, TT, and TSH values of PE patients and healthy men.

Item	Baseline	Posttreatment	Control
5-HT (ng/ml)	75.84 ± 35.1	118.69 ± 48.6^*∗*^	135 ± 40.19^*∗*^
NO (*μ*mol/l)	26.89 ± 2.18	31.59 ± 0.77*∗*	31.08 ± 2.38*∗*
Oxytocin (pg/ml)	98.79 ± 7.77	71.01 ± 13.13*∗*	67.73 ± 15.13*∗*
Prolactin (ng/ml)	7.35 ± 1.35	6.75 ± 2.5	7.22 ± 2.22
FSH (mIU/mL)	6.68 ± 1.13	6.13 ± 1.68	6.35 ± 1.49
LH (mIU/mL)	6.94 ± 1.47	6.82 ± 1.74	7.02 ± 1.21
TT (ng/ml)	5.04 ± 1.72	3.82 ± 1.21*∗*	2.93 ± 1.71*∗*
TSH (mIU/mL)	3.07 ± 1.55	2.78 ± 1.54	3.61 ± 1.79

*∗p* < 0.05, compared with baseline; 5-HT: serotonin; NO: nitric oxide; FSH: follicle-stimulating hormone; LH: luteinising hormone; TT: total testosterone; TSH: thyroid-stimulating hormone; PE: premature ejaculation.

## Data Availability

All data generated or analyzed during this study are included in this published article.

## Abbreviations

QS: Qiaoshao
TCM: Traditional Chinese medicine
PE: Premature ejaculation
PPI: Protein-protein interaction
ESS: European Society of Surgeons
IELT: Intravaginal ejaculatory latency time
FF: Forsythiae Fructus
PR: Paeoniae Radix Alba
RB: Radix Bupleuri
HM: Hedysarum multijugum Maxim.
MO: Morindae officinalis Radix
RD: Rhizoma Dioscoreae
AR: Acoritataninowii Rhizoma
OB: Oral bioavailability
DL: Drug-likeness
MCODE: Molecular complex detection
CC: Cellular component
MF: Molecular function
BP: Biological processes
NANC: Nonadrenergic noncholinergic
NO: Nitric oxide
OTR: Oxytocin receptors.

## Supplementary Materials

Supplementary Materials Figure S1: PPI network of module 1 derived from the PPI network of PE-related targets. Figure S2: PPI network of module 2 derived from the PPI network of PE-related targets. Figure S3: PPI network of module 3 derived from the PPI network of PE-related targets. Figure S4: the pathway-target network of the PE-related targets. Table S1: chemical parameters of the active compounds in QS formula. Table S2: detail information of the compound-compound targets network. Table S3: PE-related targets retrieved from databases. Table S4: detailed information of the disease PPI network. Table S5: GO analysis results of the QS formula-PE targets. Table S6: GO analysis results of the module 1 derived from the QS formula-PE targets. Table S7: GO analysis results of the module 2 derived from the QS formula-PE targets. Table S8: GO analysis results of the module 3 derived from the QS formula-PE targets. Table S9: detailed topological characteristics of gene-pathways network of PE-related 567 targets. Table S10: GO enrichment analysis results of QS formula-PE targets. Table S11: KEGG enrichment analysis results of QS formula-PE targets. Table S12: details of topological characteristics of the QS formula-PE network.
