# Supplementary figures and images for: Network Pharmacology-Based Strategy to Investigate Pharmacological Mechanisms of Qiaoshao Formula for Treatment of Premature Ejaculation
Source: Evid Based Complement Alternat Med. 2020 Nov 11;2020:1418634. doi: 10.1155/2020/1418634 (PMC7676949; doi:10.1155/2020/1418634)

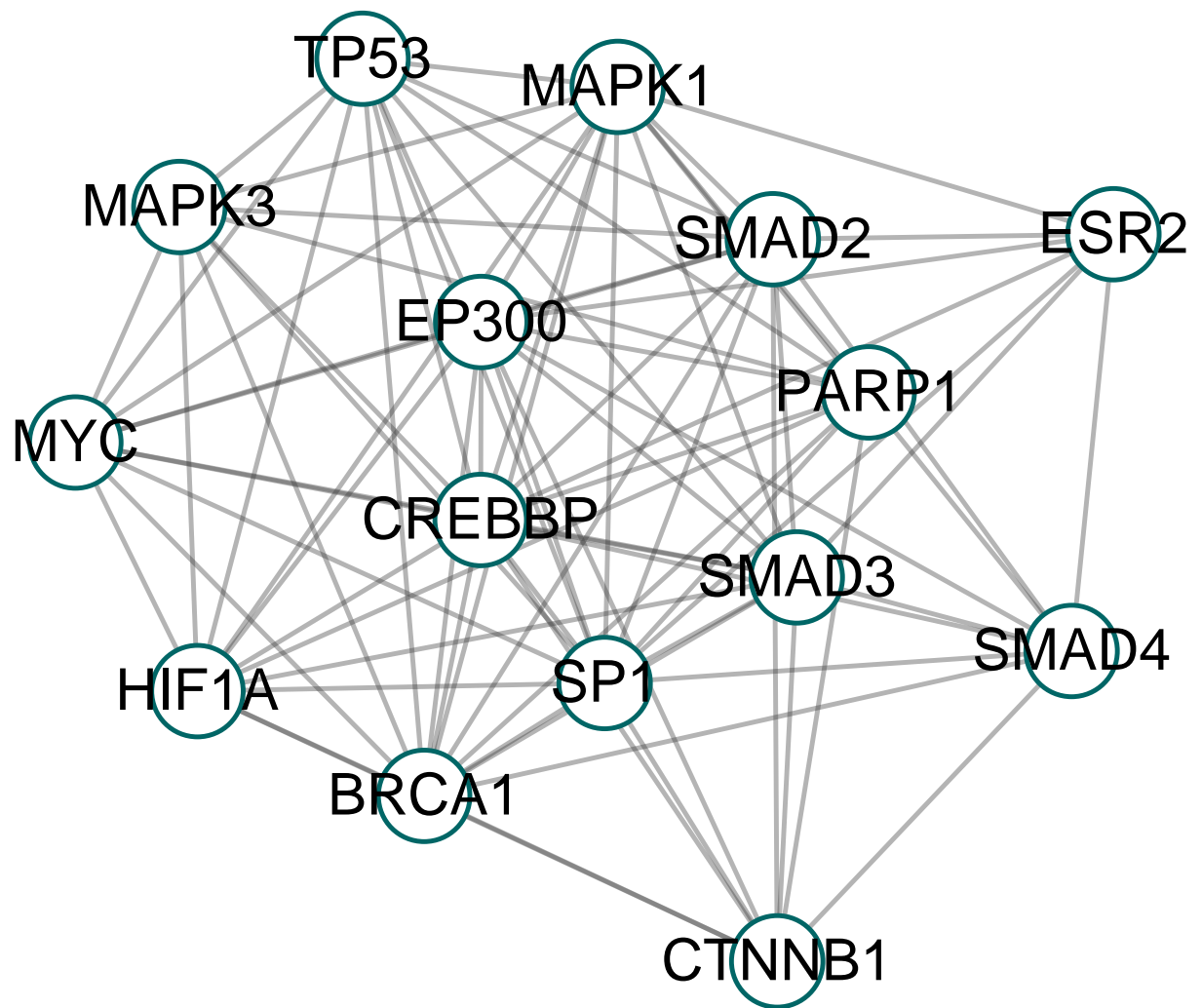

Supplement: Supplementary Materials — Figure S1: PPI network of module 1 derived from the PPI network of PE-related targets. Figure S2: PPI network of module 2 derived from the PPI network of PE-related targets. Figure S3: PPI network of module 3 derived from the PPI network of PE-related targets. Figure S4: the pathway-target network of the PE-related targets. Table S1: chemical parameters of the active compounds in QS formula. Table S2: detail information of the compound-compound targets network. Table S3: PE-related targets retrieved from databases. Table S4: detailed information of the disease PPI network. Table S5: GO analysis results of the QS formula-PE targets. Table S6: GO analysis results of the module 1 derived from the QS formula-PE targets. Table S7: GO analysis results of the module 2 derived from the QS formula-PE targets. Table S8: GO analysis results of the module 3 derived from the QS formula-PE targets. Table S9: detailed topological characteristics of gene-pathways network of PE-related 567 targets. Table S10: GO enrichment analysis results of QS formula-PE targets. Table S11: KEGG enrichment analysis results of QS formula-PE targets. Table S12: details of topological characteristics of the QS formula-PE network. [file 1418634.f1.zip › Figure S1.pdf]

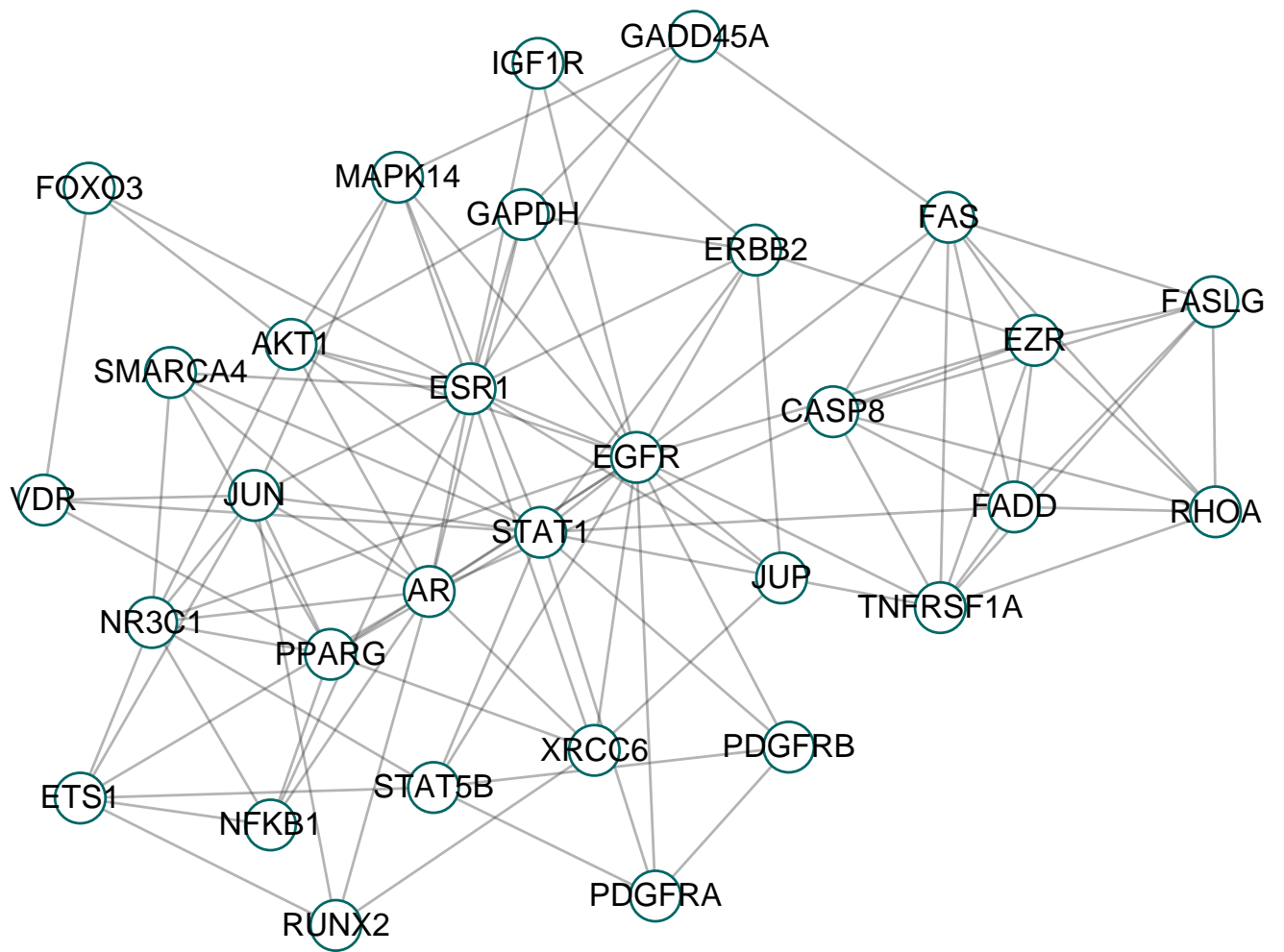

Supplement: Supplementary Materials — Figure S1: PPI network of module 1 derived from the PPI network of PE-related targets. Figure S2: PPI network of module 2 derived from the PPI network of PE-related targets. Figure S3: PPI network of module 3 derived from the PPI network of PE-related targets. Figure S4: the pathway-target network of the PE-related targets. Table S1: chemical parameters of the active compounds in QS formula. Table S2: detail information of the compound-compound targets network. Table S3: PE-related targets retrieved from databases. Table S4: detailed information of the disease PPI network. Table S5: GO analysis results of the QS formula-PE targets. Table S6: GO analysis results of the module 1 derived from the QS formula-PE targets. Table S7: GO analysis results of the module 2 derived from the QS formula-PE targets. Table S8: GO analysis results of the module 3 derived from the QS formula-PE targets. Table S9: detailed topological characteristics of gene-pathways network of PE-related 567 targets. Table S10: GO enrichment analysis results of QS formula-PE targets. Table S11: KEGG enrichment analysis results of QS formula-PE targets. Table S12: details of topological characteristics of the QS formula-PE network. [file 1418634.f1.zip › Figure S2.pdf]

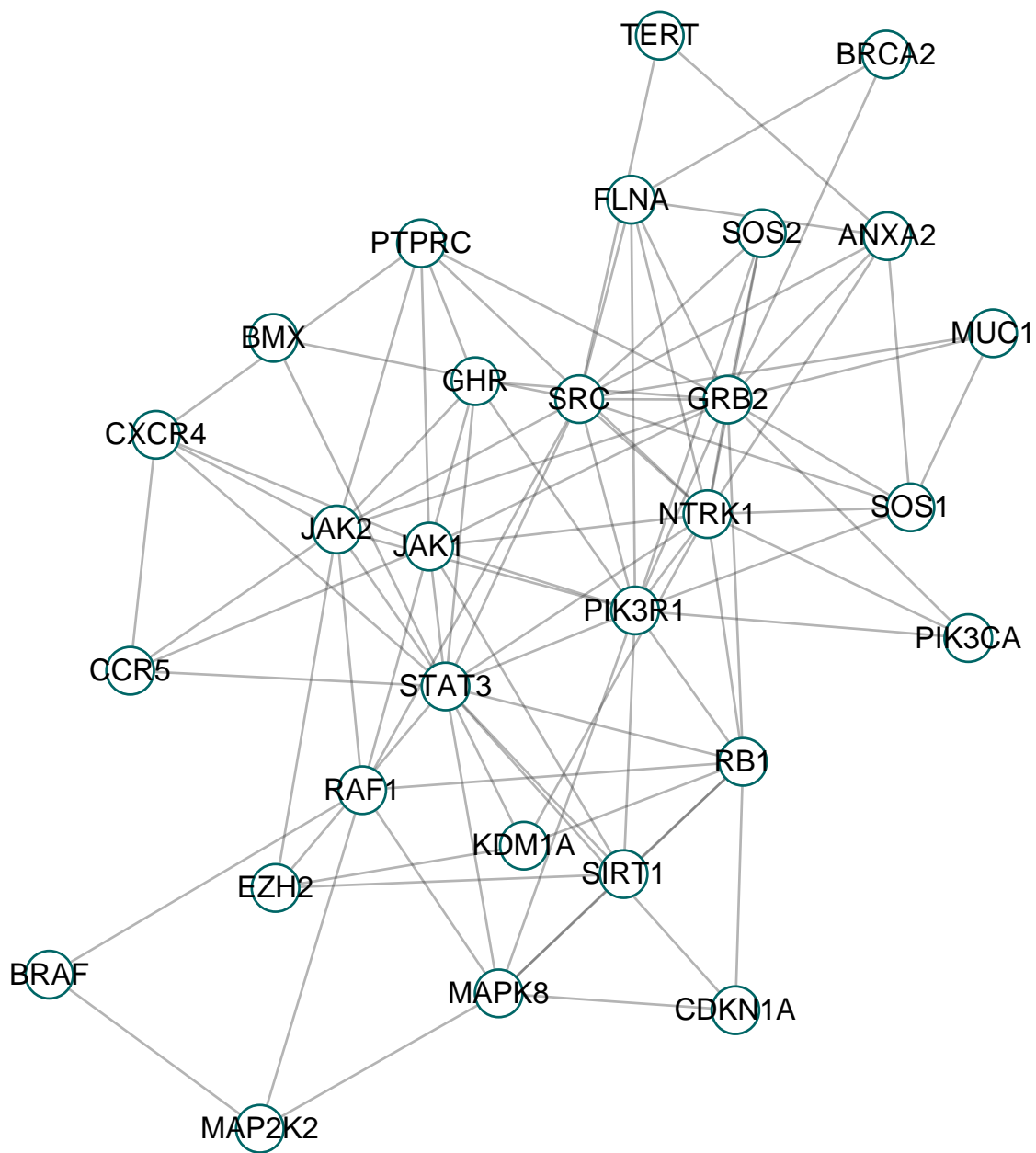

Supplement: Supplementary Materials — Figure S1: PPI network of module 1 derived from the PPI network of PE-related targets. Figure S2: PPI network of module 2 derived from the PPI network of PE-related targets. Figure S3: PPI network of module 3 derived from the PPI network of PE-related targets. Figure S4: the pathway-target network of the PE-related targets. Table S1: chemical parameters of the active compounds in QS formula. Table S2: detail information of the compound-compound targets network. Table S3: PE-related targets retrieved from databases. Table S4: detailed information of the disease PPI network. Table S5: GO analysis results of the QS formula-PE targets. Table S6: GO analysis results of the module 1 derived from the QS formula-PE targets. Table S7: GO analysis results of the module 2 derived from the QS formula-PE targets. Table S8: GO analysis results of the module 3 derived from the QS formula-PE targets. Table S9: detailed topological characteristics of gene-pathways network of PE-related 567 targets. Table S10: GO enrichment analysis results of QS formula-PE targets. Table S11: KEGG enrichment analysis results of QS formula-PE targets. Table S12: details of topological characteristics of the QS formula-PE network. [file 1418634.f1.zip › Figure S3.pdf]

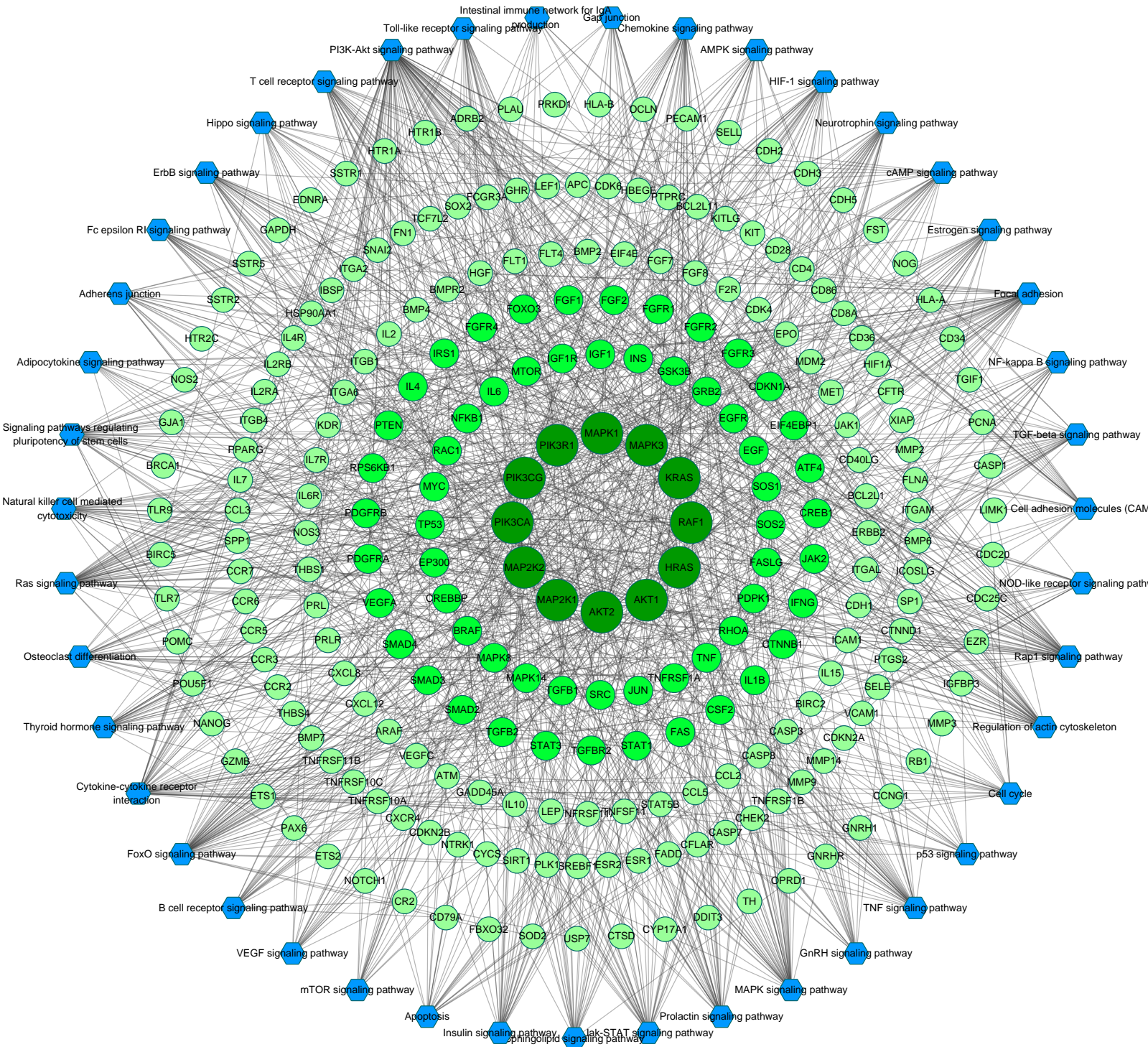

Supplement: Supplementary Materials — Figure S1: PPI network of module 1 derived from the PPI network of PE-related targets. Figure S2: PPI network of module 2 derived from the PPI network of PE-related targets. Figure S3: PPI network of module 3 derived from the PPI network of PE-related targets. Figure S4: the pathway-target network of the PE-related targets. Table S1: chemical parameters of the active compounds in QS formula. Table S2: detail information of the compound-compound targets network. Table S3: PE-related targets retrieved from databases. Table S4: detailed information of the disease PPI network. Table S5: GO analysis results of the QS formula-PE targets. Table S6: GO analysis results of the module 1 derived from the QS formula-PE targets. Table S7: GO analysis results of the module 2 derived from the QS formula-PE targets. Table S8: GO analysis results of the module 3 derived from the QS formula-PE targets. Table S9: detailed topological characteristics of gene-pathways network of PE-related 567 targets. Table S10: GO enrichment analysis results of QS formula-PE targets. Table S11: KEGG enrichment analysis results of QS formula-PE targets. Table S12: details of topological characteristics of the QS formula-PE network. [file 1418634.f1.zip › Figure S4.pdf]
